# Decoding the palliative care landscape in Nigeria: Progress, challenges, and the road ahead

**DOI:** 10.1017/S1478951525100321

**Published:** 2025-06-25

**Authors:** Tajudeen Olasinde, Tonia Onyeka, Adeniyi Adenipekun, Samuel Otene, Victoria Kajang, Olaitan Soyannwo

**Affiliations:** 1 Department of Radiation and Clinical Oncology, Ahmadu Bello University Teaching Hospital, Zaria, Nigeria; 2 Pain & Palliative Care Unit, University of Nigeria Teaching Hospital, Ituku-Ozalla, EN, Nigeria; 3 IVAN Research Institute, Nigeria; 4 Department of Radiation Oncology, University College Hospital, Ibadan, OYO, Nigeria; 5 Department of Hospice and Palliative Care, Faculty of Clinical Sciences, College of Medicine, University of Ibadan, Ibadan, Nigeria; 6 Radiology Department, Federal University of Health Sciences, Otukpo, BE, Nigeria; 7 Marie Curie Hospice, UK; 8 Palliative Care Project Unit, IAMRAT, College of Medicine, University College Hospital, Ibadan, Nigeria

**Keywords:** Palliative care, History, Progress, Challenges, Nigeria

## Abstract

**Background:**

Palliative care is a critical component of healthcare, yet its integration into Nigeria’s health system remains limited. Despite the growing burden of life-limiting illnesses, palliative care is underdeveloped, primarily restricted to tertiary institutions. This review examines the evolution of palliative care in Nigeria, key milestones, persistent challenges, and future directions for strengthening its implementation.

**Methods:**

This narrative review synthesized historical records, policy documents, and literature on palliative care in Nigeria. It examined leadership roles, institutional efforts, and government policies influencing Palliative care growth, while highlighting implementation gaps and opportunities.

**Results:**

Palliative care in Nigeria has evolved from early grassroots efforts to structured institutional services. Key milestones include the establishment of the Hospice and Palliative Care Association of Nigeria (HPCAN), and policy advancements such as the National Policy and Strategic Plan for Hospice and Palliative Care. Despite these developments, challenges persist, including inadequate funding, workforce shortages, limited opioid access, policy implementation gaps, and socio-cultural barriers. Leadership engagement, targeted policy advocacy, and comprehensive capacity-building are essential to overcoming these barriers.

**Significance of Results:**

Sustained efforts are needed to fully integrate palliative care into Nigeria’s healthcare system. Strategic interventions, including enhanced policy implementation, funding mechanisms, workforce development, and community engagement, are critical for ensuring equitable access to PC services. Strengthening collaborations between the government, healthcare institutions, and international partners will accelerate progress, ultimately improving the quality of life for patients with life-limiting illnesses.

## Background

Palliative care (PC) is a form of medical management whose definition has evolved over time from being a specialty that aims at improving the quality of life of adult and pediatric patients with chronic life-limiting illnesses and that of their families and caregivers, to being an active, culturally sensitive, holistic care to improve the quality of life for all individuals of all ages experiencing serious health-related suffering (SHS) and provide emotional, mental, and other support for their families and caregivers (Elessi 2021; Radbruch et al. 2020b). It addresses their physical, emotional, psychosocial, and spiritual needs, such as the relief from pain and other distressing symptoms, irrespective of the stage of the illness or the need for curative treatment. PC encompasses the continuum of care from diagnosis of a serious illness to treatment and cure for some patients or hospice, end-of-life care, and post-bereavement care in relation to the terminally ill individual (Cain et al. 2018). It is also care that is individualized, patient-focused, family support-oriented, affirming life, not hastening death but uplifting symptom relief at its essence (Radbruch et al. 2020a; Spencer et al. 2019), qualities unrivalled by any other medical specialty. Patients receive PC together with curative treatments such as chemotherapy, radiation, surgery, and pain management. In addition, it has been found to be less costly and more cost-effective compared to standard care with no palliative care focus.

Globally, PC is recognized as a critical component of comprehensive healthcare systems, yet its accessibility and integration vary significantly. PC is essentially an ignored global health priority as it is still virtually non-existent in most places in the world despite the growing burden of both communicable and non-communicable disease (Peeler et al. 2024; Radbruch et al. 2020a). About 80% of individuals in need of PC live in low- and middle-income countries (LMICs). Even with the 2014 World Health Assembly Resolution 67.19, which introduced a stand-alone resolution titled “Strengthening Palliative Care as a Component of Comprehensive Care Throughout the Life Course,” significant progress in global PC development remains limited a decade after its adoption. The resolution highlighted the critical need for integrating palliative care into health systems at all levels, recognizing it as an essential component of universal health coverage (UHC). It called on member states to take actionable steps, such as formulating national policies, improving access to essential PC medicines (including opioids for pain relief), and strengthening training and education for healthcare providers. Despite these directives, implementation has faced numerous challenges. Many countries, especially in LMIC regions, still lack robust PC policies, adequate funding, and access to essential medications. The shortage of trained PC professionals and the persistence of cultural and systemic barriers have further slowed progress. Moreover, inequities in access to PC persist, with a disproportionate burden of suffering falling on vulnerable populations in resource-limited settings. These gaps highlight the urgent need for renewed global and local commitments to translate the resolution’s vision into meaningful actions, ensuring that individuals across the life course have access to compassionate and comprehensive PC.

Nigeria’s healthcare system operates within a three-tier framework, comprising primary, secondary, and tertiary care, with services provided by both public and private sectors. Despite being Africa’s most populous country, the healthcare system is underfunded, with significant disparities in access to care across urban and rural areas. PC services, which are essential for managing the burden of life-limiting illnesses like cancer, sickle cell disease, HIV/AIDS, and end-of life issues in all age groups, are underdeveloped and restricted to a few tertiary care facilities, hence the classification of Nigeria as having “isolated PC services.” The lack of integration of PC into the broader healthcare system, coupled with limited trained personnel and inadequate access to pain management resources, underscores the urgent need for strategic interventions to address this growing need. This review seeks to address these challenges by examining the development and progression of PC in Nigeria within the broader context of global health and local healthcare systems. It aims to illuminate key milestones and achievements that have shaped PC delivery in the country, while critically analyzing the persistent challenges that hinder its full integration into the Nigerian healthcare framework. Additionally, the review seeks to provide insights into future directions and opportunities for advancing PC in Nigeria, emphasizing strategies for addressing barriers, leveraging local and international collaborations, and aligning efforts with global PC standards to meet the growing PC needs of Nigerian patients and their families.

## Evolution of PC in Nigeria

### Early efforts, milestones, and state of affairs

The roots of hospice and palliative care in Nigeria can be traced back to the early 1970s when Dame Cicely Saunders widely regarded as the founder of modern-day hospice care, visited St. Luke’s Hospital in Wusasa, Zaria, Kaduna State, during a missionary outreach program. During her visit, she rendered PC services and introduced the concept of hospice care, planting the seeds for what would become a transformative movement in Nigeria’s healthcare landscape. The next significant milestone occurred in 1993 when Mrs. O.K. Fatunmbi, a Nigerian nurse with a passion for compassionate care, received specialized training in hospice care at St. Christopher’s Hospice in the United Kingdom and met Dr. Anne Merriman, the founder of Hospice Africa Uganda. Inspired by her training and encouraged by Anne, she pioneered Nigeria’s first home-based PC initiative while a nurse at the University Teaching Hospital (LUTH) with Professor J.T.K Duncan and later on her retirement a home-based palliative care service in Lagos, thus introducing a model that brought comfort and dignity to patients and their families in their homes. Building on this foundation, Mrs. Fatunmbi, alongside her husband and other visionaries, joined forces with Professor J.T.K. Duncan, a radiation oncologist, who served as Chairman of the Board of Trustees, to establish Hospice Nigeria in 1998. Registered as a charity and non-profit organization, Hospice Nigeria became a trailblazer in delivering hospice and PC services in Lagos State, setting the stage for a more structured and organized approach to addressing the needs of patients with life-limiting illnesses in later years.

In a significant leap forward for the hospice and palliative care movement in Nigeria, the Hospice and Palliative Care Association of Nigeria (HPCAN) was founded on March 28, 2007, by Professor Anne Merriman during a visit and at a palliative care workshop at National Hospital, Abuja. Her encouragement and a seed fund of $200 served as a catalyst for the association’s formation. Officially registered with Nigeria’s Corporate Affairs Commission (CAC), HPCAN became the umbrella body for hospice and PC providers in Nigeria, protecting the rights of patients and their families as well as fostering collaboration, advocacy, and professional development within the field. Key milestones in Nigeria’s PC history before the advent of HPCAN include the establishment of the first hospital-based PC unit at the Federal Medical Centre, Abeokuta, Ogun State (Oliver et al. 2011), and the first hospital-based Hospice and PC unit with home-based service at the University College Hospital (UCH), Ibadan in Oyo State, in 2007. The UCH team started from 1996 with heightened advocacy for opioid availability, accessibility for effective pain management especially pain due to advanced cancer in the country (Adenipekun et al. 2005). These institutions have played a pioneering role in providing comprehensive, patient-centered care for individuals with life-limiting illnesses, serving as models for integrating PC into hospital settings across the country. HPCAN began with an initial membership of just 20 individuals but has grown exponentially to include over 165 individual and corporate members nationwide. This growth reflects the increasing recognition of the critical role PC plays in enhancing the quality of life for patients and their families. Today, HPCAN continues to champion the development and integration of PC into Nigeria’s healthcare system, building on the foundational efforts of early pioneers and ensuring that compassionate care remains at the heart of medical practice and government healthcare policy in the country.

### Role of key stakeholders

In the early stages of PC development in Nigeria, individual stakeholders such as Dame Cicely Saunders and Professor Anne Merriman played catalytic roles, laying the groundwork for the introduction of the concept of PC to Nigerian healthcare providers. Decades later, Mrs. Fatunmbi would build on that foundation, demonstrating the feasibility of community-based models. Private institutions have also made significant contributions to the development of PC in Nigeria. The expansion of PC services has been further supported by private cancer facilities like Lakeshore Cancer Center, which offers palliative and supportive care services in Lagos State and its surrounding areas. The Heart of Gold Hospice, located at Surulere, Lagos, opened on October 2, 2023, and is the only pediatric stand-alone hospice in Nigeria, catering for children with varying forms and levels of disabilities. The Centre for Palliative Care, Nigeria (CPCN), a non-governmental and non-profit organization based in Ibadan, Oyo State, was established in October 2005. It has a multidisciplinary team that is dedicated to providing comprehensive hospice and PC services to patients, while also advancing education and research in the field. In collaboration with management of UCH, it developed a unit of Hospice and Palliative Care (Adenipekun et al. 2005). By 2016, the Unit became a full-fledged department of Hospice and Palliative Care. CPCN has intensified its efforts to include PC in the revised Bachelor of Medicine, Bachelor of Surgery (MBBS) curriculum of University of Ibadan. It has continued its strong advocacy for PC in the University of Ibadan through the College of Medicine for establishment of an Academic Department for Hospice and Palliative Care. It’s leadership collaborated with the Cancer Desk Officer at the Federal Ministry of Health, Nigeria (FMoH), to sponsor health professionals for a five-week PC course at Hospice Africa Uganda and later ran the course in Ibadan, training professionals from Nigeria’s six geopolitical zones. CPCN also facilitated “start-up” PC workshops in several tertiary institutions. A collaboration with MD Anderson Cancer Center (2009–2017) enabled Nigerian health professionals to undergo clinical attachments. CPCN also partnered with the American Cancer Society’s through its Treat the Pain project, which, in collaboration with the FMoH, led to the local production of oral morphine from morphine powder in Nigeria (O’Brien et al. 2018).

### Achievements of HPCAN

The continued establishment and expansion of interest in PC in public institutions in Nigeria is a landmark achievement of HPCAN and a reflection of the association’s unwavering dedication to promoting equitable access to quality care for patients with life-limiting illnesses. HPCAN has played a pivotal role in advocating for and catalyzing the integration of PC services within Nigeria’s healthcare system, particularly in public institutions. The presence of PC units and interested health professionals in major federal tertiary hospitals across Nigeria has further strengthened the country’s palliative care infrastructure. These fully operational units, offering in-patient and outpatient services, are currently located in institutions such as the Federal Medical Centre, Abeokuta, University College Hospital, Ibadan, University of Ilorin Teaching Hospital, University of Nigeria Teaching Hospital, Enugu, Ahmadu Bello University Teaching Hospital, Zaria, and several others across the nation (see Table 1). Collectively, both public and private institutions have been instrumental in the ongoing development and accessibility of palliative care services in Nigeria. These institutions, through their dedicated services and support, have significantly contributed to the improvement of quality of life for patients with life-limiting illnesses. On its part, the Federal Government of Nigeria, in collaboration with successive pioneers and leadership of HPCAN, has recognized the vital role of PC in the healthcare system and has taken substantial steps to promote its integration. A major milestone was the dedication of a section of the Nigeria National Cancer Control Plan (NCCP) 2018–2022, to the policy on Hospice and PC, advocating for the integration of PC services within oncology treatment centers. This was a critical move toward ensuring that cancer patients and those with other life-limiting diseases have access to holistic care that addresses not only their medical needs but also their emotional and spiritual well-being. Building on this foundation, the government has recently formalized its commitment to PC by introducing the stand-alone National Policy and Strategic Plan for Hospice and Palliative Care (2021). This policy serves as a comprehensive framework to guide the development of PC services across the country, ensuring that it becomes an integral part of Nigeria’s healthcare delivery system and is accessible to all who need it. The association also contributed to the development of the National Policy for Controlled Medicines (NPCM) and its implementation strategies which advocate use of controlled drugs for medical and scientific purposes, and the rational prescription and dispensing of these medications by qualified health personnel, while preventing diversion and abuse (Federal Ministry of Health N 2017). HPCAN’s collaborations with global organizations such as the International Association for Hospice and Palliative Care (IAHPC), African Palliative Care Association (APCA), American Cancer Society (ACS), Global Partners In Care (GPIC), and the End-of-Life Nursing Education Consortium (ELNEC) have provided technical and financial support for the integration of PC into Nigeria’s public healthcare system. These partnerships have facilitated the implementation of best practices and the adaptation of global guidelines to local contexts.

**Table 1.** Locations of private and public palliative care practices in Nigeria by region

Region	Institution
South East	University of Nigeria Teaching Hospital, Enugu, Enugu State; Federal Medical Centre, Umuahia, Abia State;
South South	University of Port Harcourt Teaching Hospital, Port Harcourt, Rivers State; *Rivers State University Teaching Hospital, Port Harcourt, Rivers State;
South West	Federal Medical Centre, Abeokuta, Ogun State; Olabisi Onabanjo University, Sagamu, Ogun State; *University of Lagos Teaching Hospital, Lagos, Lagos State; University College Hospital, Ibadan, Oyo State; Centre for Palliative Care, Nigeria, Ibadan, Oyo State; Lakeshore Cancer Centre, Lagos, Lagos State; Centrepoint Medical, Lagos, Lagos State; **Heart of Gold Hospice, Lagos, Lagos State;
North Central	National Hospital Abuja, Federal Capital Territory (FCT); *University of Abuja Teaching Hospital, Gwagwalada, Abuja, FCT; St Anne’s Hospice, Gwagwalada- Abuja, FCT; Federal Medical Centre, Keffi, Kebbi State; *Federal Medical Centre, Lokoja, Kogi State; Federal Medical Centre, Makurdi, Benue State.
North East	Modibbo Adama University Teaching Hospital, Yola, Adamawa State; *Federal Teaching Hospital, Gombe, Gombe State;
North West	Ahmadu Bello University Teaching Hospital, Kaduna, Kaduna State;

* New centers from 2023.

** Pediatric-only Hospice.

HPCAN has maintained a rich tradition of fostering knowledge sharing, collaboration, and professional growth through its Annual Scientific Conferences and Annual General Meetings (AGMs). The inaugural Scientific Conference and AGM took place in 2008 in Ibadan, marking a significant milestone in the organization’s efforts to promote PC as an integral component of Nigeria’s healthcare system. This tradition continued with successive conferences in Zaria (2009), Enugu (2010), Abuja (2011), Ilorin (2012), Port Harcourt (2013), Oshogbo (2014), Ilishan (2015), Makurdi (2016), Umuahia (2017), and Yola (2018). In 2019, HPCAN returned to Makurdi for another impactful conference, but the global COVID-19 pandemic brought unprecedented challenges in 2020, forcing the cancellation of that year’s event. Undeterred, HPCAN adapted by adopting a hybrid format for its conferences starting in 2021, allowing both in-person and virtual participation to ensure continuity and inclusivity. The hybrid conferences began in Zaria (2021), followed by Ibadan (2022), Enugu (2023), and Port Harcourt (2024). As HPCAN continues to expand its reach and influence, Lagos is set to host the 2025 conference in July, promising another landmark event in the association’s history. These conferences serve as a beacon for advancing knowledge, practice, and policy in PC. Regarding the current PC educational landscape, efforts have been made by HPCAN through its members in their respective institutions to ensure the inclusion of PC content in the undergraduate medical curriculum (Onyeka et al. 2013; Tolulope and Buchanan 2024) in three universities – namely, the University of Ibadan, the University of Ilorin, and the University of Nigeria – a nursing elective at Bayero University, Kano, and some PC components within the postgraduate medical curriculum for the Diploma in Family Medicine program (National Postgraduate Medical College of Nigeria 2021), thereby laying a foundation for equipping future healthcare professionals with the necessary knowledge and skills to provide quality PC. Presently, the association is working with the National Postgraduate College of Nigeria to produce a Fellowship training program for PC through the Family Medicine specialty. Working alongside Society for the Study of Pain, Nigeria (SSPN) and the Federal Ministry of Health in the latter’s partnership with the ACS, the association has ensured the availability of immediate-release liquid oral morphine compounded at and dispensed from select tertiary hospitals in the country. HPCAN’s journey has been shaped and guided by the exemplary leadership of its Board of Trustees and past presidents, whose vision and dedication have steered the organization toward excellence and impact. Their contributions have been pivotal in expanding membership, increasing public awareness, and fostering collaboration within the PC community in Nigeria and beyond. The association remains committed to its mission of enhancing the quality of life for patients and families facing life-limiting illnesses, with its conferences serving as a beacon for advancing knowledge, practice, and policy in PC.

## Systemic challenges in PC care delivery

### Policy implementation challenges

The integration of PC into Nigeria’s healthcare system has been significantly hindered by several policy gaps and implementation challenges. Despite the establishment of the National Policy and Strategic Plan for Hospice and Palliative Care, there has been a notable lack of effective implementation, leading to a disconnect between policy intentions and practical application. This gap is further exacerbated by the absence of hospital-based PC guidelines, resulting in inconsistent practices and a lack of standardized care across healthcare facilities. Efforts to promote the policy have predominantly relied on passive dissemination methods, such as distributing informational materials, which have proven insufficient in fostering active engagement and commitment among healthcare providers. Studies suggest that more proactive strategies, including leadership engagement, policy advocacy, and targeted capacity-building, are essential to overcome these implementation barriers. However, challenges such as inadequate administrative buy-in, fragmented policy dissemination, and a lack of resources for implementation continue to impede the operationalization of PC policies. These barriers contribute to the underutilization and underdevelopment of PC services, leaving many patients without access to essential end-of-life care (Agom et al. 2020). Furthermore, the integration of PC within Nigeria’s health system faces additional obstacles, including cultural influences, denial or rejection of diagnosis by patients, and inappropriate attitudes of healthcare workers. Addressing these challenges requires a comprehensive approach that includes policy reform, education, and the development of clear, actionable guidelines to ensure the effective delivery of PC services (Ndiok and Ncama 2021).

### Funding constraints and human resource limitations

PC in Nigeria faces significant funding constraints that impede its development and accessibility. Government funding for PC programs remains limited (no provision for PC in the national budget), leading to a heavy reliance on out-of-pocket expenses for patients. This financial burden is exacerbated in the management of cancer patients who often forego essential treatments due to cost prohibitions. Additionally, palliative care medications are not included in Nigeria’s National Health Insurance Scheme (NHIS), further exacerbating the financial challenges faced by patients and their families. The exclusion of these critical medications from the NHIS means that patients must bear the full cost, which is often unaffordable (Ogbenna et al. 2022). The lack of adequate funding also hampers infrastructure development, including the establishment of dedicated PC wards and office spaces. Without proper facilities, providing quality PC becomes exceedingly difficult. Moreover, the scarcity of financial resources impedes manpower development, limiting opportunities for healthcare professionals to attend conferences or pursue further education, such as bachelor’s or master’s degrees in PC. This lack of professional development opportunities is important because it contributes to a workforce that may not be fully equipped to meet the complex needs of patients requiring PC, especially as it has been noted that there is a low level of general knowledge of PC among health professionals in Nigeria (Bastos et al. 2024). In addition, with the lack of established PC training programs for healthcare providers in the country, PC physicians and other team members often run the risk of developing burnout which would grossly impair their clinical judgments. Efforts to secure donor funding and encourage private-sector involvement have been challenging. The absence of a robust funding framework and the lack of awareness about the importance of PC among potential donors and private investors have hindered the mobilization of necessary resources. Consequently, PC services remain underfunded, affecting their quality and availability. Addressing these funding constraints is crucial for the development of a sustainable and effective PC system in Nigeria (Agom et al. 2021).

**Analgesic supply chain gaps**: Choice of analgesic agents continues to be very limited. Oral morphine stock-outs in the country are a perennial problem. Opioids like morphine are only available to few urban tertiary medical institutions, leaving the vast majority of hospitals (primary care and secondary care facilities) in the country without. While restrictions still remain largely in place for its lawful prescribing by physicians only, the National Policy for Controlled Medicines (Federal Ministry of Health N 2017) and its Implementation Strategies mandate that in the absence of a doctor, licensed Community Health Officer (CHO), Community Health Extension Worker (CHEW) or Junior Community Health Extension Worker (JCHEW) can prescribe such medicines at the Primary Health Centres, Primary Health Clinics, and Health Posts.

### Cultural and societal barriers

Cultural and societal barriers significantly impede the effective delivery of PC in Nigeria. A prevalent misconception equates PC solely with end-of-life services, leading to its underutilization by patients who might benefit from early integration of supportive care. This misunderstanding is compounded by the stigma associated with terminal illnesses and with cancer and HIV/AIDS, which fosters societal discrimination and discourages individuals from seeking timely medical intervention. Such stigmatization not only isolates patients but also exacerbates their psychological distress, as evidenced by studies indicating high levels of anxiety and depression among Nigerian cancer patients (Akin-Odanye and Husman 2021; Eguzo et al. 2022; Eseadi and Amedu 2024). Additionally, the term “palliative” in Nigeria is presently colloquially misinterpreted to mean food relief or humanitarian aid, an assumption with its origins from the COVID-19 peri-pandemic period. This semantic confusion further obscures the true purpose of PC, hindering public understanding and acceptance. Efforts to address these challenges have been limited, with existing initiatives primarily focusing on passive dissemination of information rather than active engagement and education. Research suggests that overcoming these barriers requires robust leadership engagement, targeted policy advocacy, and comprehensive capacity-building to effectively translate policy into practice (Agom et al. 2021; Ogbenna et al. 2022).

## The road ahead: opportunities and strategies

### Policy and advocacy

Strengthening the policy framework for PC in Nigeria remains a critical priority. The existing National Policy on PC, though not yet fully implemented across the country, remains a critical framework for advancing PC in Nigeria. However, it requires regular updates to align with global best practices while being tailored to address the unique local needs and healthcare challenges. HPCAN remains committed to intensifying its advocacy efforts to ensure the policy’s full implementation. Additionally, the association will continue to advocate for dedicated budgetary allocations for PC services within the health sector, recognizing the importance of sustained funding in achieving equitable access to quality PC nationwide. A sustained collaboration with policymakers, non-governmental organizations (NGOs), and international bodies with an interest in palliative care can significantly enhance efforts to strengthen PC services in Nigeria. Integrating PC into universal health coverage (UHC) on a global scale and ensuring the inclusion of essential PC medications within the NHIS on a local scale are pivotal steps toward its growth and development. These measures would not only make PC services more accessible and affordable for patients but also help establish a sustainable framework for integrating PC into the broader healthcare system.

### Education and capacity building

Expanding education and training opportunities for healthcare providers is key to addressing the current shortage of PC specialists in Nigeria. Over the years, HPCAN has consistently advocated for establishing structured curricula on PC in medical, nursing, and allied health schools, because this can ensure early exposure and skill development. Continuous professional development (CPD) programs, which focus on training healthcare workers in pain management, communication skills, and psychosocial support, are also very important tools that could help bring healthcare workers up to speed on PC practices. Developing comprehensive frameworks for CPD programs is not only feasible but also essential for sustaining and expanding the capacity of PC providers in Nigeria. These frameworks should focus on leveraging technology for remote learning, fostering partnerships and collaborations with academic institutions and international organizations, and tailoring training content to meet the unique challenges faced in the Nigerian healthcare context. By prioritizing CPD programs, HPCAN can equip existing and emerging clinicians with the necessary skills to deliver high-quality PC, thereby strengthening the PC workforce and improving access to essential services across the country. In addition, HPCAN urges its members to pursue postgraduate studies at the newly approved academic department for Hospice and Palliative Care at the University of Ibadan, and encourages other Nigerian universities to work toward similar goals in order to increase PC training opportunities for healthcare professionals in Nigeria. Education and training for caregivers especially for patient support at home are also essential since the traditional extended family network has been eroded by urbanization and family members’ migration away from the country.

### Sustainable funding

Funding PC programs sustainably requires innovative approaches. Partnerships with private sector players, philanthropists, and international donors may help complement government funding. Establishing public–private partnerships (PPPs) can also provide a steady financial base for hospice operations, and advocating for community-based health insurance schemes and social health programs to incorporate PC would enable affordability for patients. Advocacy campaigns targeting corporate social responsibility (CSR) initiatives could help raise funds, while also increasing awareness of palliative care needs.

### Community engagement and cultural sensitivity

The role of community engagement in ensuring that PC services are accessible and acceptable cannot be overemphasized. Therefore, it is important to build partnerships with community leaders, religious groups, and traditional healers. This can effectively foster trust and collaboration. Culturally sensitive approaches that respect local beliefs and traditions must be integrated into service delivery. Public awareness campaigns tailored to diverse audiences including faith-based organizations can be used to dispel misconceptions about PC, such as its consistent association solely with end-of-life care and misconceptions about death and dying. Encouraging community involvement in volunteer programs can also create a supportive network for patients and their families.

### Leveraging technology

Technology offers immense potential to bridge the existing gaps in PC access. Telemedicine platforms can be utilized to extend PC consultations to remote and underserved areas, reducing barriers to care (Van Gurp et al. 2015). Digital tools, such as mobile apps and e-learning platforms, can enhance education and training for healthcare providers and caregivers in the country. Research in these areas using Nigerian patient and informal caregiver populations is ongoing, and data collection and analysis systems continue to support evidence-based decision-making and improve PC service delivery (Namisango et al. 2024; Nkhoma et al. 2021). Moreover, leveraging social media and online platforms for advocacy and awareness can amplify the reach of PC campaigns.

## Conclusion

The progress made in developing PC in Nigeria demonstrates significant achievements, including the establishment of structured PC services in public institutions and inclusion of PC in the National Cancer Control Plan and the National Policy and Strategic Plan for Hospice and Palliative Care. The leadership of the HPCAN has been instrumental in advancing these milestones through advocacy, capacity building, and fostering collaborations with global partners. Despite these advancements, challenges such as limited funding, inadequate integration into healthcare systems, cultural misconceptions, and a shortage of trained personnel persist. Addressing these barriers is crucial to ensuring that PC becomes accessible to all Nigerians, particularly those in underserved communities.

Looking forward, a strategic roadmap that prioritizes policy advocacy, sustainable funding, workforce development, and leveraging technology is essential to building a robust PC system in Nigeria. By fostering partnerships with stakeholders at all levels, working against the global backdrop of universal health coverage PC integration and inclusion of PC medication in the Nigerian National Health Insurance Scheme, the nation can make significant strides in addressing the growing PC needs of its population. With continued commitment from HPCAN and other stakeholders, the vision of equitable access to quality PC across Nigeria is achievable, ensuring dignity and improved quality of life for all individuals with life-limiting illnesses.
